# Selection for environmental variance of litter size in rabbits

**DOI:** 10.1186/s12711-017-0323-4

**Published:** 2017-05-22

**Authors:** Agustín Blasco, Marina Martínez-Álvaro, Maria-Luz García, Noelia Ibáñez-Escriche, María-José Argente

**Affiliations:** 1 Institute for Animal Science and Technology, Universitat Politècnica de València, Valencia, Spain; 2 Departamento de Tecnología Agroalimentaria, Universidad Miguel Hernández de Elche, Orihuela, Spain; 3 Genètica i Millora Animal, Institut de Recerca i Tecnologia Agroalimentàries, Caldes de Montbui, Spain

## Abstract

**Background:**

In recent years, there has been an increasing interest in the genetic determination of environmental variance. In the case of litter size, environmental variance can be related to the capacity of animals to adapt to new environmental conditions, which can improve animal welfare.

**Results:**

We developed a ten-generation divergent selection experiment on environmental variance. We selected one line of rabbits for litter size homogeneity and one line for litter size heterogeneity by measuring intra-doe phenotypic variance. We proved that environmental variance of litter size is genetically determined and can be modified by selection. Response to selection was 4.5% of the original environmental variance per generation. Litter size was consistently higher in the Low line than in the High line during the entire experiment.

**Conclusions:**

We conclude that environmental variance of litter size is genetically determined based on the results of our divergent selection experiment. This has implications for animal welfare, since animals that cope better with their environment have better welfare than more sensitive animals. We also conclude that selection for reduced environmental variance of litter size does not depress litter size.

## Background

In recent years, there has been increasing interest in the genetic determination of environmental variance. The reasons are summarized by Morgante et al. [1] and Sørensen et al. [2]. In evolutionary genetics, how phenotypic variance is maintained under several models of selection is a key issue. For example, Zhang and Hill [3] examined models for maintenance of environmental variance under stabilizing selection, in which phenotypes near the optimum are selected and, consequently, less variable genotypes are favored. In medical genetics, there are several foci of interest, such as differences in the penetrance of risk alleles [1] or the evolution of health indicators over time [2]. In animal and plant genetics, selection to reduce environmental variance can lead to more uniform products without compromising future genetic progress, since genetic variance of the trait is not affected [4]. In addition, genetic uniformity can be useful for production traits; for example, homogeneity of birth weight within litters in rabbits is related to higher viability of the kits [5].

In the case of litter size, which is a trait directly related to fitness, environmental variance can be related to the capacity of animals to cope with new environmental conditions. Females with less adaptable genotypes are more sensitive to diseases and to stress and show a higher degree of variability in litter size [6–8]. Selection to reduce environmental variance would produce animals that cope better with their environment, which is a definition of animal welfare [9].

There is evidence that environmental variance is under genetic control in several species. Most of this evidence is indirect, because it comes from analyses of databases and not from experiments designed to assess the genetic determination of environmental variance (litter size in sheep [10] and pigs [11], birth weight and stillbirth in pigs [12], weight in snails [13], birth weight in mice [14], uterine capacity in rabbits [15], weight in poultry [16] and beef cattle [17], milk yield of dairy cattle [18], and weight in trout [19] and salmon [20]). Other evidence of the existence of a genetic component for environmental variance comes from a few experiments on inbred lines of *Drosophila melanogaster* [1] and from only two selection experiments on birth weight, in rabbits [21] and mice [22]. Models used to analyze environmental variance were reviewed by Hill and Mulder [23]. They are highly parametrized and not robust; for example, Yang et al. [24] showed that small deviations from normality in the residuals can substantially change estimates of genetic parameters.

In the experiment reported in this paper, we avoided the use of complex models of environmental variance by directly selecting for this trait as an observed trait. Environmental variance of litter size can be directly recorded by computing the intra-doe variance of litter size. Since the genetic determination of litter size is approximately the same for all parities of a rabbit doe and permanent effects are the same along parities [25], the intra-doe phenotypic variance represents the environmental variance if no other systematic environmental effects are acting. We developed a divergent selection experiment on intra-doe phenotypic variance as a measure of environmental variance of litter size.

## Methods

### Animals

The rabbits used in this study came from a maternal synthetic line created from commercial crossbred animals [26]. The rabbits were bred at the farm of the Universidad Miguel Hernández of Elche. Reproduction was organized in discrete generations. Does were first mated at 18 weeks of age and thereafter 10 days after parturition. They were under a constant photoperiod of 16:8 h and controlled ventilation. The animals were fed a standard commercial diet. All experimental procedures were approved by the Committee of Ethics and Animal Welfare of the Miguel Hernández University, according to Council Directives 98/58/EC and 2010/63/EU.

### Selection for environmental variance

**Fig. 1:**
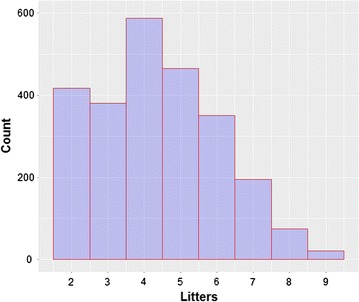
Distribution of number of litters per doe

A divergent selection experiment on environmental variance of litter size was carried out across 10 generations. Each divergent line had approximately 125 females and 25 males per generation. Data from 12,174 litters from 2769 does were used in the experiment. The average number of litters per doe was 4.5, ranging from 2 to 9 (Fig. 1).

Selection was based on environmental variance of litter size, $V_{e}$, which was calculated as the within-doe variance of litter size after litter size was pre-corrected for year-season and three levels of parity-lactation status: first parity, other parities for lactating females, and other parities for non-lactating females, to avoid systematic effects that could affect the variance. The intra-doe phenotypic variance represents the environmental variance for litter size under the assumption that the genetic determination is approximately the same for all parities of a rabbit doe and the permanent effects are the same across parities [25]. Variance $V_{e}$ for each doe was calculated using the minimum quadratic risk estimator:

$$V_{e} = \frac{1}{n + 1}\mathop \sum \limits_{1}^{n} \left( {x_{i} - \bar{x}} \right)^{2} ,$$

where $x_{i}$ is the pre-corrected litter size of a doe’s parity *i*, and *n* is the total number of parities of the doe (*n* varying from 2 to 9). This estimator has lower risk (lower expected mean square error) than that of maximum likelihood (ML) or restricted maximum likelihood (REML) [27]. The environmental variance of litter size without pre-correction was also calculated.

All dams were ranked based on their estimate of intra-doe variance of litter size, without using pedigree information for genetic evaluation. Only dams with four or more parities were considered for selection. Selection candidates came from females that had four or five parities, with some exceptions. The best 20% dams were used to breed the next generation. Each sire was mated with five dams and one male progeny from the best dam that a sire was mated to was selected to breed the next generation. This within-male family selection was performed in order to reduce inbreeding. Selection was based on the individual record of each female.

### Statistical analysis

Response to selection was estimated as the difference between lines in each generation. These differences between lines were analyzed using a simple linear model with a line-generation effect:

$${\mathbf{y}} = {\mathbf{Xb}} + \varepsilon ,$$

where **y** is a vector with one record per doe, i.e. its environmental variance $V_{e}$, and **b** is a vector of the line-generation effect. This linear model has heterogeneous variances, because not all does had the same number of litter size records, so $V_{e}$ is calculated based on different numbers of parities. The weights for taking this into account were calculated as [28]:

$$\frac{{2\left( {n - 1} \right)}}{{\left( {n + 1} \right)^{2} }}\sigma_{\varepsilon }^{2} ,$$

where *n* is the number of parities of each doe and $\sigma_{\varepsilon }^{2}$ the residual variance. To check the robustness of the model, the same analysis was performed with homogeneous variances, which led to the same results with small variations in the confidence intervals.

Response to selection was also estimated as the average of the genetic values in each generation by using a mixed model with generation as a fixed effect and the breeding value of each doe as a random effect. Breeding values were assumed normally distributed with variance ${\mathbf{A}}\sigma_{u}^{2} ,$ where **A** is the pedigree-based relationship matrix and $\sigma_{u}^{2}$ is the variance of the breeding values. In this model, the generation effect captures systematic changes in environment over generations.

Correlated response in litter size was estimated as the differences in litter size between lines in each generation. It was analyzed using a standard mixed model with fixed effects of line-generation, parity-lactation status (first parity, and lactating or not at mating in other parities) and year-season, along with a random permanent environmental effect across parities for each doe, which was assumed normally distributed.

Bayesian analyses were performed to fit the above models, with bounded flat priors for all unknowns. Features of the marginal posterior distributions were estimated using Gibbs sampling. After some exploratory analyses, we used a chain of 60,000 samples for differences between lines with a burn-in period of 10,000; only one of every 10 samples was saved for inferences. For the genetic analyses, we used a chain of 1,000,000 samples, with a burn-in of 500,000; only one of every 100 samples was saved for inferences. Convergence was tested using the Z criterion of Geweke [29], and Monte Carlo sampling errors were computed using time-series procedures, as described in [30]. In all Bayesian analyses, the Monte Carlo standard errors were small and lack of convergence was not detected by the Geweke test. Special software code was developed for analyses of differences between lines and the program TM was used for the genetic analyses [31].

## Results

### Descriptive results

**Fig. 2:**
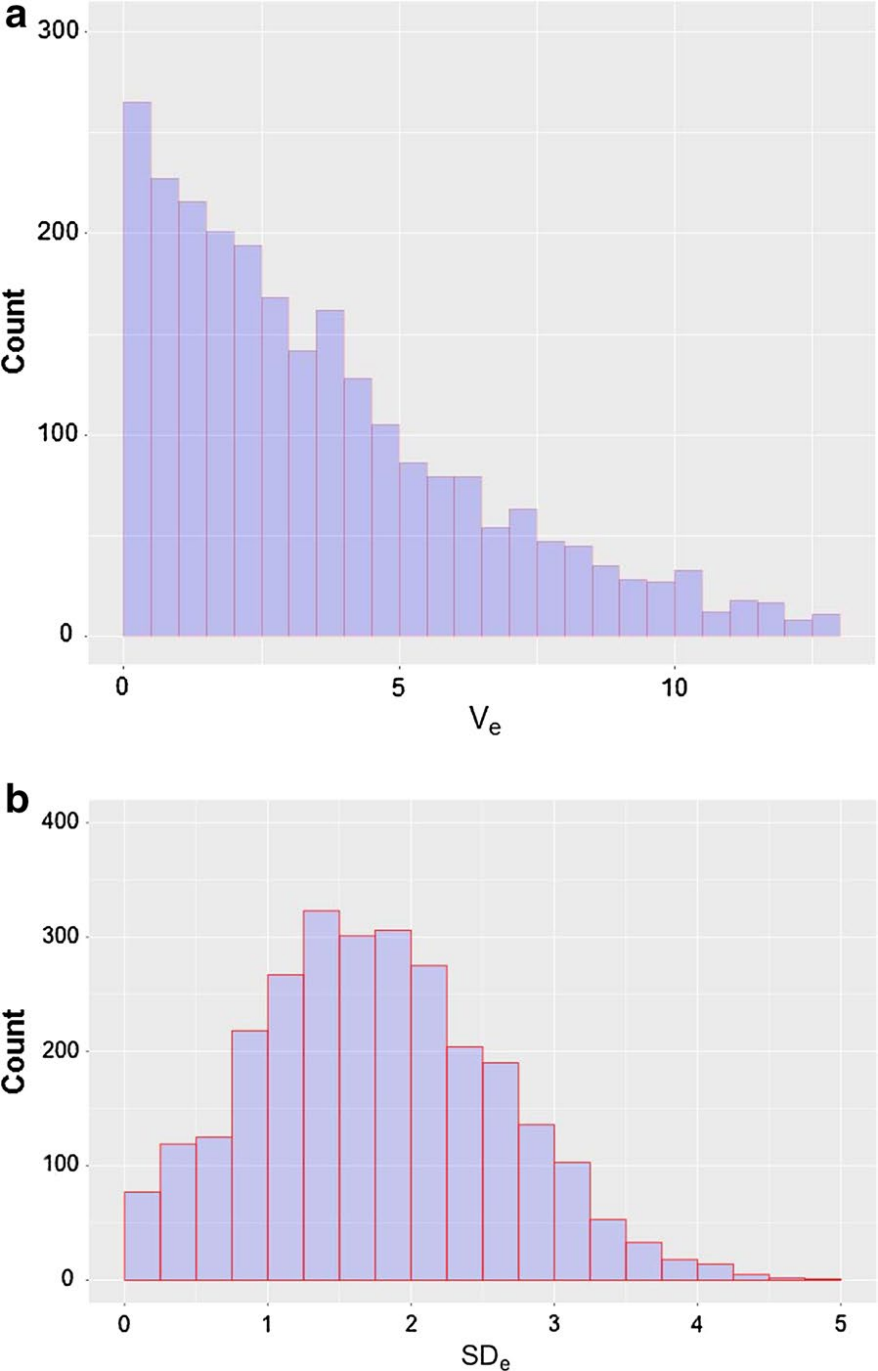
Distribution of environmental variance and standard deviation of litter size. **a** Distribution of the litter size environmental variance, $V_{e}$ (kits^2^), **b** distribution of the litter size environmental standard deviation, $SD_{e}$ (kits). Both are calculated with pre-corrected data

Table 1 summarizes the descriptive features of the traits in the base population. We estimated intra-doe phenotypic variance by pre-correcting for the effects of year-season and parity-lactation status (first parity, and lactating or not at mating in all subsequent parities). Pre-correction had little effect with environmental variance before ($V_{r}$) and after pre-correction ($V_{e}$) being practically the same. In both cases, environmental variances were highly variable, with a large standard deviation and high coefficient of variation, which helps explain the large response to selection, which will be presented below. The median of the environmental variance differs from its mean, showing that its distribution is asymmetrical, as expected (Fig. 2a). Although normality is not required for comparison of means when the sample size is moderate or large, we applied a normalizing transformation to the environmental variance. We chose the square root because it has a biological interpretation, i.e. environmental standard deviation ($SD_{e}$). For this trait, the mean and median were similar (Table 1; Fig. 2b). The distribution of the number of parities per doe is in Fig. 1.

**Table 1:** Descriptive statistics of the evaluated traits in the base population

	Mean	Median	SD	CV
$V_{e}$	3.73	2.72	3.36	0.90
$V_{r}$	3.96	3.13	3.55	0.90
$SD_{e}$	1.74	1.65	0.84	0.48
$LS$	8.71	9.00	3.01	0.35

SD, standard deviation; CV, coefficient of variation; $V_{e}$, environmental variance of litter size based on pre-corrected data; $V_{r}$, environmental variance of litter size based on uncorrected data; $SD_{e}$, environmental standard deviation of litter size based on pre-corrected data; $LS$, litter size

### Response to selection

Response to selection was high and equal to approximately 4.5% of the mean of the environmental variance per generation. In generation 10, response to selection was 1.67 kits^2^, which is 45% of the original mean, with a 95% confidence interval of [0.85, 2.53]. In a Bayesian context, several confidence intervals can be easily estimated. We can provide intervals [k, +∞), where k can be interpreted as a guaranteed value with a determined probability [32]. The guaranteed value of the environmental variance at 80% probability was 1.32 kits^2^, which means that the response was at least 1.32 kits^2^ with 80% probability. When the environmental variance was taken without pre-correcting data, the response in generation 10 was 1.74 kits^2^, with a 95% confidence interval of [0.88, 2.61], and a guaranteed value at 80% probability of 1.36 kits^2^, showing that pre-correction had a small effect. The average standard deviation ($SD_{e}$) had a response of 0.46 kits in generation 10, with a guaranteed value of 0.36 kits at 80% probability.

**Fig. 3:**
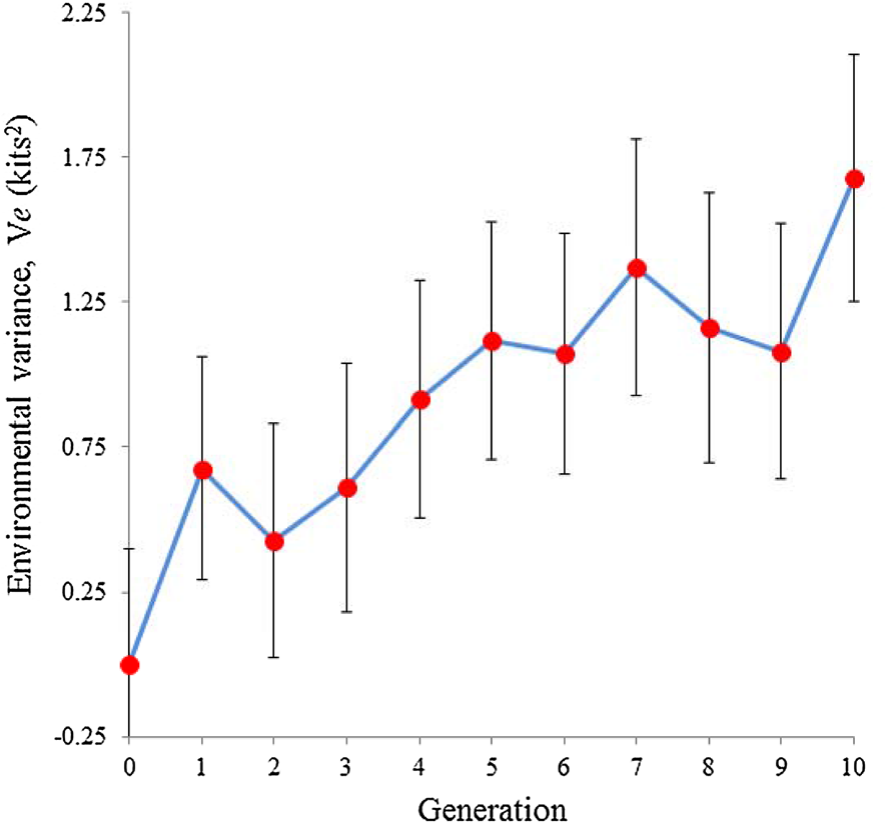
Response to selection for environmental variance of litter size. Differences between the High and Low lines for environmental variance of litter size calculated with pre-corrected data, $V_{e}$. The means and standard deviations of the marginal posterior distributions of the difference between lines are plotted for each generation. *Bars* represent the standard deviation of the posterior distribution of the differences

**Fig. 4:**
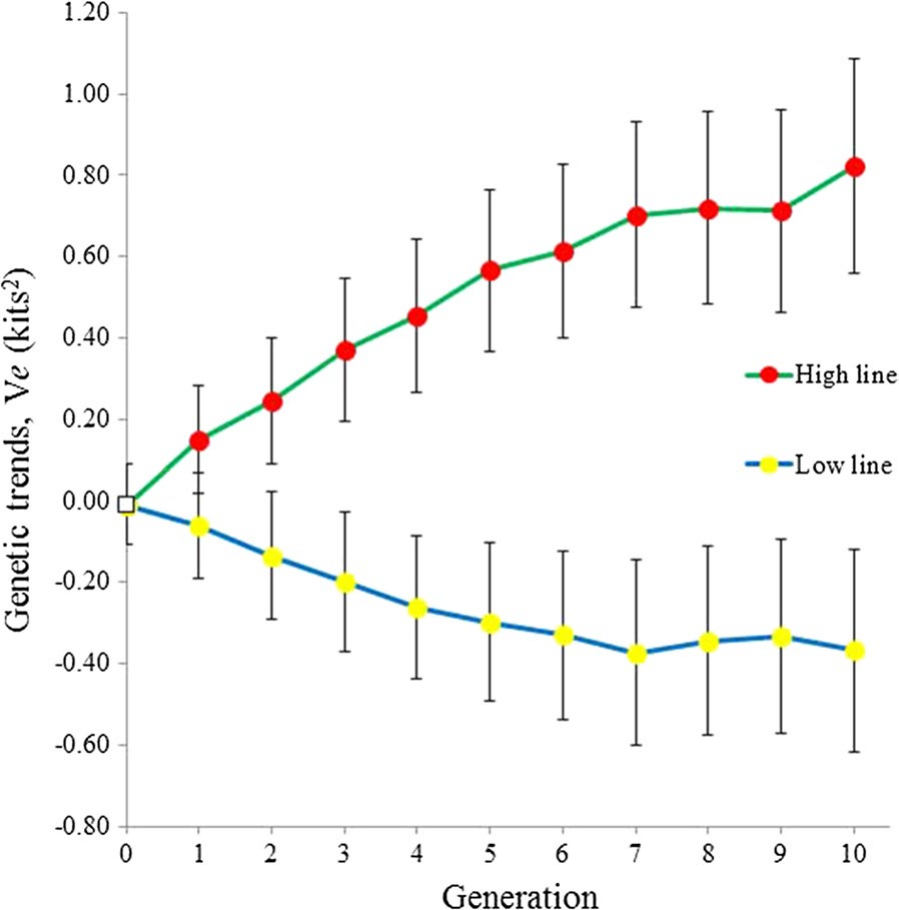
Response to selection for environmental variance of litter size in the High and Low lines. Genetic means per generation of the litter size environmental variance calculated with pre-corrected data, $V_{e}$. *Bars* represent the standard deviation of the posterior distribution of the genetic means

For each generation, the mean and standard deviation of the marginal posterior distributions of the differences between the High and Low lines are plotted in Fig. 3. Response to selection was higher in the first generation, likely due to the higher selection pressure applied (Table 2). In divergent selection experiments, the number of animals in the base generation is twice the size of each divergent line, and greater selection pressures can be applied. Response to selection in each line, which is derived from the estimated genetic means in each generation, is shown in Fig. 4, with the corresponding standard deviations of the posterior distributions. Selection appeared to be more successful in increasing environmental variance than in decreasing it, which agrees with the lower selection differentials that could be applied in the Low line (Table 2). The differences in genetic means between lines are consistent with the phenotypic differences found in Fig. 3, which corroborates the model used.

**Table 2:** Weighted selection differentials for $V_{e}$ (kits^2^) by generation

	High line	Low line
Base population	3.0	1.5
Generation 1	1.5	0.2
Generation 2	1.7	0.3
Generation 3	2.9	0.6
Generation 4	1.8	0.2
Generation 5	2.0	0.9
Generation 6	2.4	1.0
Generation 7	2.9	0.8
Generation 8	1.7	0.2
Generation 9	2.4	0.4

### Correlated response in litter size

**Fig. 5:**
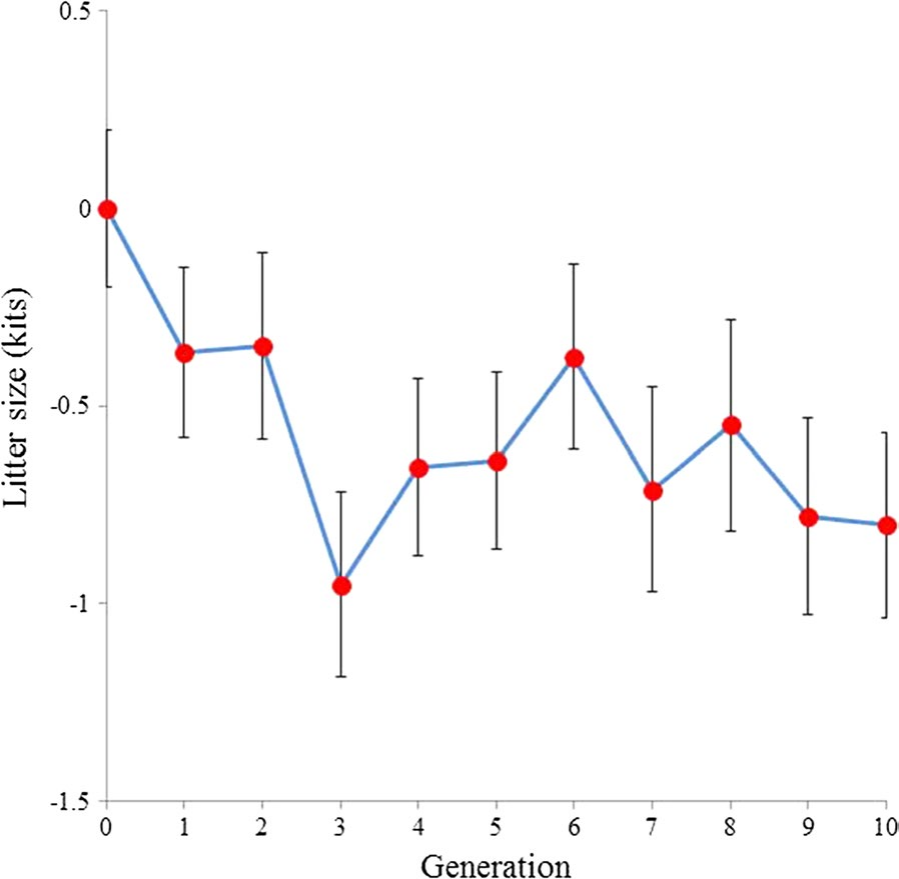
Correlated response to selection in litter size. Differences in litter size between the High and Low lines. Means and standard deviations of the marginal posterior distributions of the difference between lines are plotted for each generation. *Bars* represent the standard deviation of the posterior distribution of the differences

Litter size was consistently larger in the Low line than in the High line throughout the experiment (Fig. 5). In the last generation of selection, the difference in litter size between the Low and High lines was 0.80 kits, with a 95% confidence interval of [0.34, 1.26] and a guaranteed value of 0.60 kits at 80% probability and 0.41 kits at 95% probability.

### Genetic parameters

Heritabilities and genetic correlations of $V_{r}$ and $LS$ with $V_{e}$ are in Table 3. The heritability of $LS$ was low, as expected, but the heritability of $V_{e}$ was also low; thus, the response to selection in $V_{e}$ that was obtained should be attributed to its large variability (Table 1). The genetic correlation between litter size variance before and after pre-correction was near 1, which indicates that the impact of pre-correction on the genetic determination of this trait was small. The genetic correlation between $V_{e}$ and $LS$ was almost null, which indicates that selection for homogeneity does not reduce litter size.

**Table 3:** Genetic parameters

	${\text{h}}^{2}$	HPD95%	${\text{r}}_{\text{g}}$	HPD95%	${\text{r}}_{\text{p}}$	HPD95%
$V_{e}$	0.08	0.05, 0.11				
$V_{r}$	0.09	0.05, 0.13	0.99	0.97, 1.00	0.97	0.967, 0.972
$LS$	0.10	0.08, 0.13	−0.06	−0.31, 0.21	−0.09	−0.14, −0.03

${\text{h}}^{2}$, heritability; HPD95%, high posterior density interval at 95%; ${\text{r}}_{\text{g}}$, genetic correlation with $V_{e}$; ${\text{r}}_{\text{p}}$, phenotypic correlation with $V_{e}$; $V_{e}$, environmental variance of litter size based on pre-corrected data; $V_{r}$, environmental variance of litter size based on uncorrected data; $LS$, litter size

## Discussion

There is some evidence in several species that environmental variance can be under genetic control, although only two selection experiments to investigate this have been performed, both using selection for birth weight, in mice [22] and rabbits [21]. A major problem in analyzing environmental variance comes from the complexity of the models that are often used, which are highly parametrized and have nested effects and parameters. Double hierarchical generalized linear models [18, 33, 34] using residual maximum likelihood and Bayesian nested models [11, 15] have been proposed to analyze this problem. These models are not robust, as shown by Yang et al. [24], who compared genetic parameters after a Box–Cox transformation to normalize the residuals in litter size in pigs and uterine capacity in rabbits. These authors showed that the coefficient of correlation between the trait and its residual variance changed dramatically as a result of transformation, when compared to the results on the untransformed scale, even changing sign in the case of pig litter size. Here, we used a more straightforward and robust criterion for selection, the intra-doe phenotypic variance for litter size, which was considered as the observed environmental variance for litter size. Models as simple as those currently used for other observed traits can then be used to analyze response to selection.

**Fig. 6:**
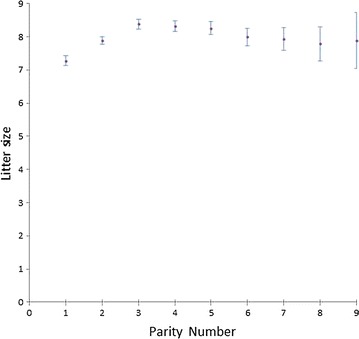
Average litter size by parity (kits)

Environmental variance of litter size was estimated as intra-doe phenotypic variance for litter size after pre-correction for season and parity-lactation status. This pre-correction was made under the hypothesis that systematic effects can affect environmental litter size variance of does; for example, a doe that has more parities during one season could have a smaller environmental variance than a doe that has parities across several seasons. The same could occur with the parity-lactation effect; it is well known that there is an effect of first parity on litter size when compared with other parities (Fig. 6); failing to consider this would cause overestimation of the environmental variance of females that have few parities. Nevertheless, in our data, these effects were so small that we would have obtained almost the same genetic response if these corrections had not been considered, since the genetic correlation between environmental variance with and without pre-corrected data was almost 1 (Table 3). Since the number of parities per doe was not large, variance estimators did not give the same result. We decided to estimate intra-doe variance using the best quadratic estimator; i.e. the one with the smallest risk.

Response to selection was estimated in two ways: as phenotypic differences between lines in each generation and as genetic trends from the estimated genetic means. All methods that are based on genetic means (best linear unbiased prediction—restricted maximum likelihood or Bayesian methods) are model-dependent, and the genetic trends depend directly on the genetic parameters used [35, 36]; for example, if the narrow-sense heritability is overestimated because dominant and epistatic components are not considered in the model, a higher genetic trend and a decreasing environmental trend will be observed. The advantage of the simple phenotypic difference between the High and Low lines is that they are independent of any model; whether there are major genes, dominance or other effects, the difference between lines is only due to genetic causes, since they were bred and raised in the same environment. When the phenotypic differences are coincident with the estimates based on a genetic model, the genetic model is corroborated (in the Popper sense [37], i.e. the model has more support for the results obtained). Conversely, the advantage of using genetic means is that we can observe the evolution of the genetic means in each line separately. Resulting responses to selection by line (Fig. 4) indicated some asymmetry in responses, with selection appearing less successful in the Low line than in the High line. There are many reasons that can explain asymmetrical response to selection (for example, Falconer and MacKay [38] list eight different reasons); here, the trend towards more homogeneity in litter size tends to reduce the possibility of high selective pressure.

The line selected for low environmental variance of litter size resulted in larger litter size in all generations than the High line. Estimating the correlation between the mean and the variance of a trait has been the goal of several studies, with various results. A negative relationship between the mean of a trait and its environmental variance was detected for litter size in pigs [11, 34], for litter size and litter weight at birth in mice [14, 39], for weight gain in mice [40], for uterine capacity in rabbits [15], and for body weight in broiler chickens [41]. By contrast, no relationship between mean and environmental variance was found for slaughter weight in pigs [42] or for birth weight in rabbits [21, 43], and a positive correlation between mean and environmental variance was found for body weight in snails [13] and broiler chickens [16] and for body conformation in broiler chickens [16]. There has been some controversy about the validity of the analyses of genetic parameters when environmental variance is estimated with highly parametrized models, such as the model of San Cristobal et al. [44]. Yang et al. [24] showed that the negative genetic correlation between uterine capacity in rabbits and its residual variance reported by Ibáñez-Escriche et al. [15] became almost null when the residuals were normalized. In our case, the estimate of the genetic correlation between $V_{e}$ and $LS$ was almost null, which agrees with the results of Yang et al. [24] for uterine capacity in rabbits, a trait that is closely related to litter size [45]. However, as we have seen, litter size was consistently larger in the Low line than in the High line throughout the experiment, which is compatible with a low negative genetic correlation within the limits of the high posterior density interval at 95% (HPD95%). The important result is that selection for homogeneity does not seem to reduce litter size of does.

The line selected for litter size homogeneity also tolerated external stressors more effectively than the line selected for litter size heterogeneity. The High line had a higher subclinical immune response, which is related to a greater sensitivity to diseases or to less tolerance to common microorganisms in the farm microenvironment [46], and after vaccination, the Low line had a quicker and higher response to invading agents [6, 7]. Response to stress was also better in the Low line; after injection of the stressing agent adrenocorticotropic hormone, the High line had a higher cortisol level, thus a higher level of stress than the Low line. The High line also showed higher hepatic activity [8]. Thus, in general, the High line was more sensitive to stress and had a lower immune response to infections. This has consequences on disease resistance but also on animal welfare, since animals that cope more effectively with their environment have better welfare than animals that are more sensitive.

## Conclusions

This is the first experiment of selection on the environmental variance of litter size and the first experiment in which selection has been directly performed on environmental variance as an observed trait. We conclude that the environmental variance of litter size is genetically determined, based on the result of our divergent selection experiment. This has consequences on animal welfare, since animals that cope better with their environment have better welfare than more sensitive animals. We also conclude that selection for reduced litter size variability does not depress litter size.
